# Modulation of cytokine patterns and microbiome during pregnancy in IBD

**DOI:** 10.1136/gutjnl-2019-318263

**Published:** 2019-06-05

**Authors:** Janine van der Giessen, Dana Binyamin, Anna Belogolovski, Sigal Frishman, Kinneret Tenenbaum-Gavish, Eran Hadar, Yoram Louzoun, Maikel Petrus Peppelenbosch, Christien Janneke van der Woude, Omry Koren, Gwenny Manel Fuhler

**Affiliations:** 1 Department of Gastroenterology and Hepatology, Erasmus MC - University Medical Center Rotterdam, Rotterdam, The Netherlands; 2 Azrieli Faculty of Medicine, Bar-Ilan University, Safed, Israel; 3 Department of Mathematics, Bar-Ilan University, Ramat-Gan, Israel; 4 Rabin Medical Center and the Sackler Faculty of Medicine, Tel Aviv university, Tel Aviv, Israel

**Keywords:** inflammatory bowel disease, crohn’s disease, ulcerative colitis, intestinal microbiology, cytokines

## Abstract

**Objective:**

Pregnancy may affect the disease course of IBD. Both pregnancy and IBD are associated with altered immunology and intestinal microbiology. However, to what extent immunological and microbial profiles are affected by pregnancy in patients with IBD remains unclear.

**Design:**

Faecal and serum samples were collected from 46 IBD patients (31 Crohn’s disease (CD) and 15 UC) and 179 healthy controls during first, second and third trimester of pregnancy, and prepregnancy and postpartum for patients with IBD. Peripheral blood cytokine profiles were determined by ELISA, and microbiome analysis was performed by sequencing the V4 region of the bacterial 16S rRNA gene.

**Results:**

Proinflammatory serum cytokine levels in patients with IBD decrease significantly on conception. Reduced interleukin (IL)-10 and IL-5 levels but increased IL-8 and interferon (IFN)γ levels compared with healthy controls were seen throughout pregnancy, but cytokine patterns remained stable during gestation. Microbial diversity in pregnant patients with IBD was reduced compared with that in healthy women, and significant differences existed between patients with UC and CD in early pregnancy. However, these microbial differences were no longer present during middle and late pregnancy. Dynamic modelling showed considerable interaction between cytokine and microbial composition.

**Conclusion:**

Serum proinflammatory cytokine levels markedly improve on conception in pregnant patients with IBD, and intestinal microbiome diversity of patients with IBD normalises during middle and late pregnancy. We thus conclude that pregnancy is safe and even potentially beneficial for patients with IBD.

**Significance of this study**

**What is already known on this subject?**

- Pregnancy constitutes a unique physiological state in which immune and microbial changes prepare the female body for fetal implantation, growth and nourishment.
- Immune dysfunction and microbial dysbiosis are common features in patients suffering from IBD.
- Some immune diseases show amelioration of disease activity during pregnancy suggesting that pregnancy-induced physiological changes affect disease pathology.
- For many patients with IBD, pregnancy is associated with uncertainties regarding disease activity and outcomes for the fetus.

**What are the new findings?**

- Proinflammatory cytokines (interleukin (IL)-6, IL-8, IL-12, IL-17 and tumour necrosis factor (TNF)α) decrease significantly on conception in patients with IBD.
- Healthy women show pregnancy-associated changes in serum cytokines during the trimesters of pregnancy that are not seen in pregnant patients with IBD.
- IBD immunological state as assessed by serum cytokine profiles are patient specific but not influenced by disease type, inflammation or pregnancy trimester.
- Both patients with UC and Crohn’s disease display a disease manifestation-specific but low-diverse microbiome before and during early pregnancy.
- IBD-associated dysbiosis as assessed by microbial diversity disappears during middle and late pregnancy.
- Cytokine and microbial network and dynamic analysis provide detailed data on correlations between microbiome, disease type, cytokine profiles and pregnancy.

**Significance of this study**

**How might it impact on clinical practice in the foreseeable future?**

- From an immunological and microbiological viewpoint, pregnancy in patients with IBD is beneficial and can be safely recommended to patients.
- As pregnancy is associated with changes of the intestinal microbiota, microbiome-directed interventions (eg, faecal transplantations, antibiotics or probiotic therapies) are not recommended in pregnancies complicated by IBD.

## Introduction

IBD, including Crohn’s disease (CD) and UC, are complex multifactorial diseases. Impaired epithelial barrier function, environmental triggers, genetic susceptibility, as well as an ineffective immune reaction towards the intestinal microbiota, contribute to a chronic intermittent intestinal inflammation.^1 2^ As IBD affects women in their reproductive years, a common concern for patients with IBD is how pregnancy will affect their disease course, and conversely, how the disease will affect their pregnancy and fetal health. These concerns are not unfounded, as active disease during conception and pregnancy has been associated with worse pregnancy outcomes,^3 4^ and children of patients with IBD are themselves at increased risk of developing IBD^5^ due to genetic risk factors as well as parental environmental and microbial factors.^6–9^ Conversely, however, the effect of pregnancy on the maternal IBD disease course is less clear. A postpartum reduction of flares has been observed in both patients with CD and UC,^10 11^ although these data were disputed by Pedersen *et al*,^12^ who found an increased risk of flares in patients with UC both during pregnancy and postpartum. Pregnancy constitutes a unique state, in which hormone-induced physiological changes prepare the body for implantation, fetal growth and parturition. Changes that take place include modulation of immune function to allow for development of a major histocompatibility complex (MHC)-mismatched fetus.^13^ Thus, maternal immune tolerance was long thought to be increased throughout pregnancy; however, it is now becoming clear that immunological states fluctuate during pregnancy to support different needs at its different stages. Successful implantation requires a proinflammatory Th1 environment at the maternal–fetal interface, which is followed by a shift towards a more tolerogenic Th2 response for the main duration of pregnancy, with again increased polarisation to a Th1 response shortly before partition.^14 15^ However, it is unclear to what extent placental immunological shifts actually translate to systemic immunological changes capable of affecting disease activity in the intestinal tract, as surprisingly few studies have investigated peripheral changes across the different trimesters of pregnancy, and those that did show conflicting results.^16–18^

In addition to immunological changes, it has been shown that the intestinal microbiome is altered during healthy pregnancy, with reduced microbial diversity observed in the third trimester, which was shown to be similar to the microbiome in patients with metabolic disease.^19^ While it is known that the microbiome and the immune system are in close reciprocal relationship,^20–23^ it is as yet unclear whether immunological and microbial changes during pregnancy are correlated. Third trimester stool microbiota was shown to harbour inflammatory characteristics, with an overall increase in Proteobacteria and Actinobacteria, and it is possible that such alterations contribute the inflammatory environment needed for parturition and prepare the maternal body for the energy demands imposed by lactation.^24^ In non-pregnant patients with IBD, alterations in microbial signatures are already present, with reduced faecal bacterial diversity, decreased presence of commensal butyrate producing bacteria (eg, *Faecalibacterium prausnitzii*) and increased abundance of Proteobacteria and Actinobacteria reported as some of the most consistent findings.^25–27^ Whether pregnancy in patients with IBD further modulates the intestinal microbiota and whether microbial changes during pregnancy are associated with diseases state are currently unknown.

While pregnancy clearly affects many physiological processes that are deregulated in IBD, remarkably little is known about immune and microbial signatures in patients with IBD during pregnancy. Here, we compared peripheral blood cytokine patterns and faecal microbiome from pregnant patients with IBD and pregnant healthy controls and show that unlike healthy controls, pregnancy in IBD is not accompanied by major changes in peripheral cytokine patterns in our cohort. Furthermore, differences in microbial diversity that are present between patients with UC and CD, and IBD versus controls disappear during pregnancy.

## Materials and methods

### Patient recruitment

Women diagnosed with IBD visiting the preconception outpatient clinic at the Erasmus MC University Medical Center, Rotterdam, between March 2014 and June 2016 were asked to donate stool and serum in the first, second and third trimester (T1–T3), where possible samples were also obtained prepregnancy and postpartum (NL47357.078.13 Dutch Medical Ethical Committee). Exclusion criteria included inability to provide consent. For patients with IBD, disease type, surgical history, age of diagnosis and age at inclusion were noted, and for each time point of sample collection, medication use, flaring of disease (as assessed by clinician based on clinical findings, faecal calprotectin and/or endoscopy) and disease activity score (Harvey Bradshaw index (HBI) for CD^28^ and Simple Clinical Colitis Activity Index (SCCAI) for UC and IBD-unclassified^29^ were noted. Healthy controls were recruited at Rabin Medical Center, Petah- Tikva, Israel (Institutional Review Board Approval number 0263-15-RMC and 0608-18-RMC) and in Clalit HMO clinics at Petah-Tikva district Israel (Approval number 0135-15-COM). Following recruitment, participants provided blood and faecal samples at T1, T2 and T3. All procedures used for collection were in accordance with National Institutes of Health Human Microbiome Project standards.^30^ All participants signed informed consent.

### Sample preparation and sequencing

DNA was extracted from 0.25 g faeces of healthy pregnant women using the Power Soil DNA Isolation Kit (MoBio, Carlsbad, USA), according to the manufacturer’s instructions and following a 2 min bead-beating step (Biospec, Bartlesville, Oklahoma, USA). From pregnant women with IBD, the bacterial DNA was extracted using PureLink Microbiome DNA Purification Kit (Invitrogen, Carlsbad, California, USA) following a 2 min bead-beating step. The V4 region of the bacterial 16S rRNA gene was amplified from the extracted DNA using the 515F and 806R barcoded primers following the Earth Microbiome Project protocol.^31^ Each PCR reaction consisted of 2 µL 515F primer (10 µM), 2 µL 806R primer (10 µM), 25 µL prime star max PCR mix (Takara, Mountain View, California, USA), 17 µL ultra-pure water and 4 µL of sample DNA. DNA amplification consisted an initial denaturing step for 3 min at 95°C followed by 30 cycles of denaturation (98°C for 10 s), annealing (55°C for 5 s) and extension (72°C for 20 s), with a final elongation step at 72°C (for 1 min). Amplicons were purified using AMPure magnetic beads (Beckman Coulter, Brea, California, USA) and DNA concentration was quantified using Qubit dsDNA HS Assay (Thermo Fisher, Bartlesville, Oklahoma, USA). Samples were then pooled at equal concentrations (50 ng/µL) and purified again using 2% E-Gel (Invitrogen). DNA fragments of the appropriate size were purified using NucleoSpin Gel and PCR Clean-up (Macherey-Nagel, Düren, Germany) and sequenced using the Illumina MiSeq platform at the Genomic Ccenter, Azrieli Faculty of Medicine, Bar-Ilan University, Israel.

### Microbiome analysis

Data analysis was performed using QIIME2.^32^ Sequence reads were demultiplexed, and sequenced reads were error-corrected by Divisive Amplicon Denoising Algorithm.^33^ A phylogenetic tree was constructed and features were assigned taxonomy using Greengenes reference database.^34^ Alpha and beta diversity measures were calculated based on a feature table with samples containing at least 5928 sequences. Richness and evenness (alpha diversity parameters) were calculated using the Faith’s Phylogenetic Diversity,^35^ Shannon’s Diversity Index and Pielou’s Evenness measures.^36^ For between sample diversity (beta diversity), weighted and unweighted UniFrac distances were calculated.^37^ Over-represented and under-represented features were identified using linear discriminant analysis effect size (LEfSe).^38^

### Normalisation

Features were merged to the genus level by averaging over all features assigned to the same genus. Given the large variation in feature values, we transformed these values to Z scores by adding a minimal value to each feature level (0.01) and calculating the 10-basis log of each value. Statistical whitening was then performed on the table by removing the average and dividing by the SD of each feature. The average of each normalised bacteria over each time point was to remove the effect of time on the samples.

### Machine learning

Unsupervised learning was performed on the normalised and merged version of the 16S rRNA feature table in order to recognise patterns in the data. Principal component analysis (PCA) was performed using Python version 3.5 and its package sklearn. A two-tailed p value of less than 0.05 was considered statistically significant. A linear support vector machine was used to classify patients with UC from patients with CD using 40 support vectors. Leave-one-out cross-validation was performed. The box constraint value was 1. More complex methods were not used to limit overfitting, given the limited number of samples.

### ELISA

Blood was collected in serum separator tubes (BD Bioscience, Mississauga, Ontario, Canada). Serum was aliquoted to avoid repeated freeze–thaw cycles and stored at −80°C until analysis. ELISA for the interleukins (IL)-4, IL-5, IL-6, IL-8, IL-9, IL-10, IL-12, IL-15, IL-17, IL-21, TNFα and IFNγ were performed using a kit according to manufacturer’s protocol (Ready-SET-Go! eBioscience, San Diego, California, USA). When insufficient serum was available, a subset of cytokines was measured. PCA of cytokine data were visualised using ClustVis.^39^ Unit variance scaling was applied to rows; singular value decomposition with imputation was used to calculate principal components. X and Y axes show principal component 1, and principal component prediction ellipses were such that a new observation from the same group will fall inside the ellipse with probability 0.95. Data distribution normality was tested by Shapiro-Wilk test. Comparison of prepregnancy cytokine levels to the individual trimesters was tested with Wilcoxon matched pairs test. Comparison of cytokine patterns over time between trimesters was performed by analysis of variance (ANOVA) (Friedman test), followed by Dunn’s multiple comparison post hoc analysis. Comparisons per time point between patients with IBD and controls or UC versus CD were analysed by Mann-Whitney test. Heat map visualisation was performed using CIMminer.^40^

### Cytokine microbial network and dynamic analysis

We developed a dynamic model by computing for each two following time points the changes in cytokine levels and in bacteria log frequencies and the tested three correlations:

- Correlation between the (log) level of bacteria at point 1 versus the change in cytokine between point 1 and point 2.
- Correlation between the cytokine level at point 1 versus the change in (log) level of bacteria between point 1 and point 2.
- Correlation between the (log) level of bacteria in point 1 versus the change in (log) level of bacteria between point 1 and point 2.

We then performed the same analysis but scrambled the bacteria. We computed the minimal p values of Pearson coefficients in the scrambled sets to be around 0.01 and thus used 0.01 as the minimal value in all comparisons. A Benjamini-Hochberg correction yielded similar results but may not be appropriate here since the data are not normally distributed.

## Results

### Characteristics of patients and controls

We first determined basic characteristics of pregnant patients with IBD and normal pregnant controls. For microbiome analysis, 46 patients and 179 controls were included (table 1). Patients with IBD and controls were of similar age at time of conception and had similar mode of delivery. IBD women used more assisted reproductive technology (p<0.0001) and were more often nulliparous (p<0.0001). Three patients with IBD used antibiotics during the third trimester for urinary tract or skin infection, with one of these three also using antibiotics during the first trimester. Stillbirth occurred in two patients in trimester 3 (T3). For cytokine levels, a subgroup of 33 patients with IBD and 40 controls was analysed (table 2). In this subgroup, IBD women were younger at time of conception (p>0.0001), had a higher body mass index (BMI) (p=0.002) and were more often nulliparous (p<0.0001) compared with controls. Mode of delivery and birth outcomes were similar between patients with IBD and controls. Specific patient characteristics are summarised in online tables S1 and S2. Of 19 patients on biologicals prior to pregnancy, 68% stopped this medication after the second trimester (T2). The number of flares (as assessed by clinician, or based on clinical findings, faecal calprotectin and/or endoscopy) during pregnancy did not vary over the course of pregnancy, nor did HBI for CD or SCCAI for UC/IBD unclassified. We concluded that our study group would allow meaningful comparisons between patients with IBD and healthy controls for immunological state and microbiome profiles.

10.1136/gutjnl-2019-318263.supp1 Supplementary data

**Table 1:** Subject characteristics (faecal samples)

	Pregnant IBD patients (n=46)	Pregnant controls (n=179)	P value
Mean age at conception in years (SD)	29.7 (3.1)	31 (4.1)	0.055
Antibiotic use during pregnancy (%)	3 (6.5)	0	<0.0001
Nulliparous (%)	37 (80.4)	86 (38.4)	<0.0001
Use of assisted reproductive technology (%)	7 (15.2)	6 (2.7)	<0.0001
Delivery (%)			0.259
Vaginal delivery	37 (84.1)	180 (81.4)	
Caesarean section	7 (15.9)	41 (18.6)	
Birth outcome (%)			
Live birth	44 (95.7)	221 (98.7)	0.168
Stillbirth	2 (4.3)	0 (0)	<0.0001
Termination	0 (0)	3 (1.3)	<0.0001

**Table 2:** Subject characteristics (serum samples)

	Pregnant patients with IBD (n=33)	Pregnant healthy controls (n=40)	P value
Mean age at conception in years (SD)	29.1 (3.5)	32.8 (3.9)	<0.0001
Median prepregnancy BMI (IQR)	24.7 (22.7–27.2)	21.6 (19.5–23.7)	0.002
Nulliparous (%)	26 (78.8)	15 (37.5)	<0.0001
Use of assisted reproductive technology (%)	4 (12.1)	2 (5)	0.27
Delivery (%)			0.663
Vaginal delivery	25 (75.8)	32 (80)	
Caesarean section	8 (24.2)	8 (20)	
Birth outcome (%)			0.360
Live birth	33 (100)	39 (97.5)	
Termination	0 (0)	1 (2.5)	
Mean gestational age in weeks (SD)	36.3 (8.4)	39.1 (1.2)	0.104
Birth weight in grams (SD)	3158 (567)	3230 (397)	0.748

BMI, body mass index.

### Proinflammatory cytokine levels decrease on conception in patients with IBD and are stable and patient-specific during pregnancy

**Figure 1:**
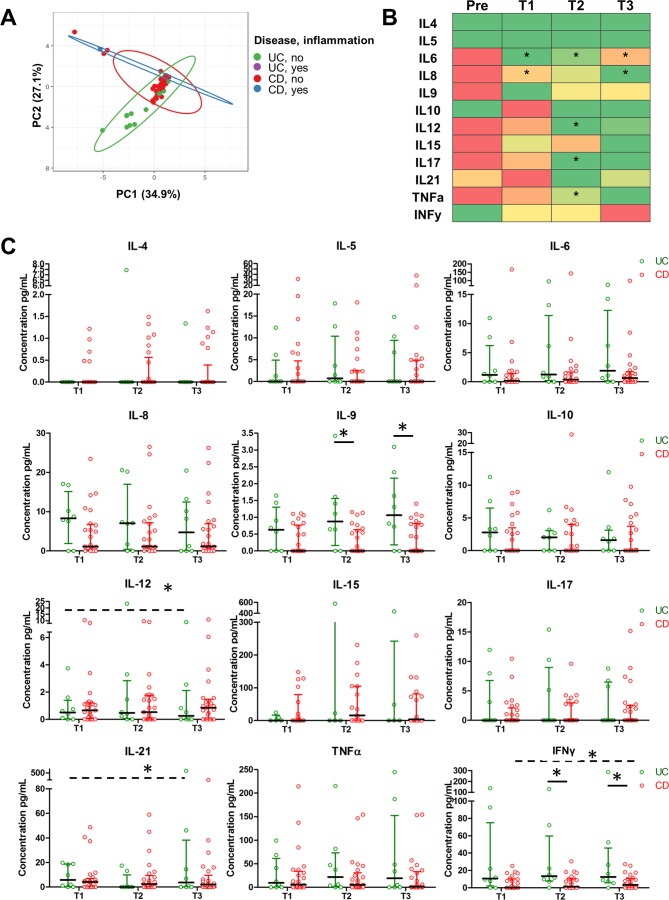
Proinflammatory cytokine serum levels decrease on conception and are stable over time during pregnancy in patients with UC and CD. (A) Principal coordinate analysis shows no overall cytokine changes during pregnancy between patients with UC patients and CD. (B) Median levels of individual cytokines are represented by a colour gradient with green indicating the lowest value for during pregnancy and red indicating the highest value. Significant decreases in several proinflammatory cytokine levels are observed in pregnancy as compared with preconception (indicated by asterisk). With the exception of TNFα, similar significant decreases were seen when patients with CD were analysed separately (not shown). (C) Comparisons of individual cytokines between patients with CD and UC for the three trimesters. Median and IQR are shown. Significant differences (Friedman test, indicated by dashed lines) for different trimesters were observed for patients with UC for IL-12 and IL-21. Significant differences between patients with UC and CD in a given trimester were seen for IL-9 and IFNγ (Mann-Whitney test, indicated by solid lines). CD, Crohn’s disease; IL, interleukin.

While altered serum cytokine patterns have been described for non-pregnant patients with IBD, it is as yet unknown whether these patterns are modulated by pregnancy in patients with IBD. We therefore first compared IL-4, IL-5, IL-6, IL-8, IL-9, IL-10, IL-12, IL-15, IL-17, IL-21, TNFα and IFNγ levels in serum obtained preconception and during the three trimesters of pregnancy in a group of 16 patients with IBD for whom paired samples were available (12 patients with CD and patients with 4 UC). PCA analysis of these samples demonstrated that overall cytokine profiles were very similar between patients with CD and UC, with samples taken during inflammation not clustering separately (figure 1A). We subsequently investigated levels of individual cytokines during pregnancy as compared with prepregnancy. Figure 1B shows that serum levels of IL-6 (p=0.005, p=0.027, p=0.012 for T1–3, respectively), IL-8 (p=0.042 for T1, p=0.012 for T3), IL-12 (p=0.034 for T2), IL-17 (p=0.0078 for T2) and TNFα (p=0.039 for T2) decreased significantly on conception. In contrast, IL-10 increased from prepregnancy to T1, although not significantly.

Next, we further explored modulation of cytokine profiles in patients with IBD during the three trimesters of pregnancy in a larger group of patients (25 CD, 8 UC, for all measurements, see online figure S1). Only IL-12 and IL-21 for patients with UC, and IFNγ for patients with CD showed modest modulation during the different trimesters of pregnancy (figure 1C, p=0.0375, p=0.0469 and p=0.0302, respectively). C reactive protein (CRP) levels increased during pregnancy (online supplementary figure S1), confirming earlier reports suggesting that CRP cannot be reliably used as a disease activity marker during late pregnancy.^40^ Direct comparisons between patients with CD and UC indicated lower IL-9 and IFNγ levels in patients with CD as compared with patients with UC in the second (p=0.0272 and p=0.0201) and third trimester (p=0.0361 and p=0.0388).

10.1136/gutjnl-2019-318263.supp2 Supplementary data

Medication use changes during pregnancy. Most noticeably, anti-TNFα use is decreased in T3, with more patients using no medication at all at this time point (online supplementary figure S2A). Usage of 5-aminosalicylic acid (ASA) and thiopurines did not fluctuate over time. PCA indicates that different treatments are not associated with an overall altered cytokine profile (online supplementary figure S2B). We next investigated whether treatment affects individual serum cytokine levels. ANOVA showed no overall differences between the different treatment regimes in terms of cytokine expression (online figure S2C). However when compared only with unmedicated patients, patients on 5-ASA showed lower IL-10 (p=0.0267) and IL-17 (p=0.0428) levels. Patients on anti-TNFα single treatment showed significantly lower IL-8 levels as compared with untreated patients (p=0.001). None of the patients stopping anti-TNFα in our cohort experienced a flare. Overall, cluster analysis showed that grouping of samples was more dependent on the individual patients rather than the disease manifestation, the presence or absence of intestinal inflammation and the pregnancy trimester from which the sample was obtained or the medication used (online supplementary figure S3). Thus, serum proinflammatory cytokine levels (and hence overall immunological state) in patients with IBD decrease on conception and are relatively stable during pregnancy.

10.1136/gutjnl-2019-318263.supp3 Supplementary data

10.1136/gutjnl-2019-318263.supp4 Supplementary data

### IL-10 and IL-6 cytokine levels increase over time in healthy pregnancies but not IBD

**Figure 2:**
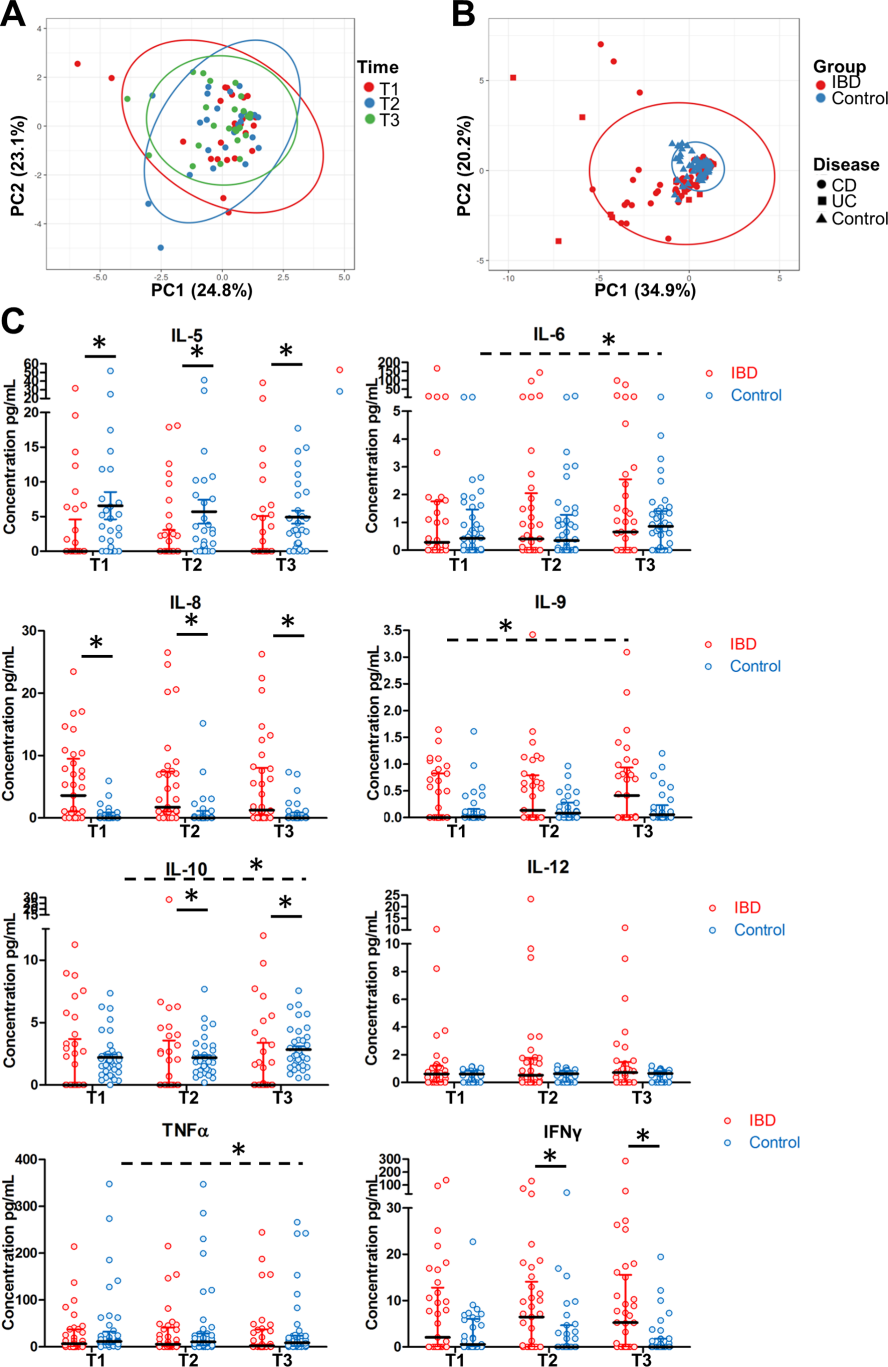
IL-6 and IL-10 serum levels rise during pregnancy in healthy controls but not patients with IBD. (A) Principal coordinate analysis (PCA) showing lack of overall cytokine changes during healthy pregnancy. (B) PCA showing that overall cytokine patterns do not cluster separately between patients with IBD and controls. Samples from first, second and third trimester were included in the analysis. (C) Comparisons of individual cytokines between patients with IBD and healthy controls for all three trimesters. Median and IQR are shown. Significant differences (Friedman test, indicated by dashed lines) for different trimesters were observed in healthy controls for IL-6, IL-10 and TNF-α and for IL-9 in patients with IBD. Significant differences between patients with IBD and healthy controls in a given trimester were seen for IL-5, IL-8, IL-10 and IFNγ (Mann-Whitney test, indicated by solid lines). IL, interleukin.

Next, we investigated whether serum cytokine levels behave differently in patients wtih IBD and healthy controls during pregnancy. PCA indicated that overall cytokine profiles did not shift over time in healthy pregnancy (figure 2A) and that overall cytokine profiles in this panel were similar when comparing patients with IBD and controls (figure 2B). However, analysis of individual cytokines showed that serum levels of IL-6, IL-10 and TNFα changed significantly over the three trimesters in healthy pregnant women (figure 2C, p=0.0124, p=0.0458 and p=0.0030, respectively). Most noticeably, both IL-6 and IL-10 levels showed a significant upregulation towards the third trimester in the healthy controls. However, this upregulation was not seen in pregnant patients with IBD, with IL-10 levels lagging significantly behind healthy controls in the second and third trimester (p=0.0385 and p=0.0016, respectively). In addition, significantly reduced IL-5 levels were observed in patients with IBD during the entire pregnancy (p=0.0194 for T1, p=0.0368 for T2, p=0.0228 for T3). In contrast, IL-8 and IFNγ levels were increased as compared with controls (IL-8: p<0.0001, p=0.0002 and p=0.0003 for T1 and T3, IFNγ: p=0.0443 for T2, p=0.0130 for T3). Patients with IBD only showed overall differences in IL-9 levels throughout pregnancy (p=0.0326). Thus, pregnancy in healthy women is associated with specific changes in peripheral blood cytokines that seem to be largely absent in expecting patients with IBD.

### The microbiome of pregnant women with IBD differs between patients with CD and UC and is affected by disease location in CD

**Figure 3:**
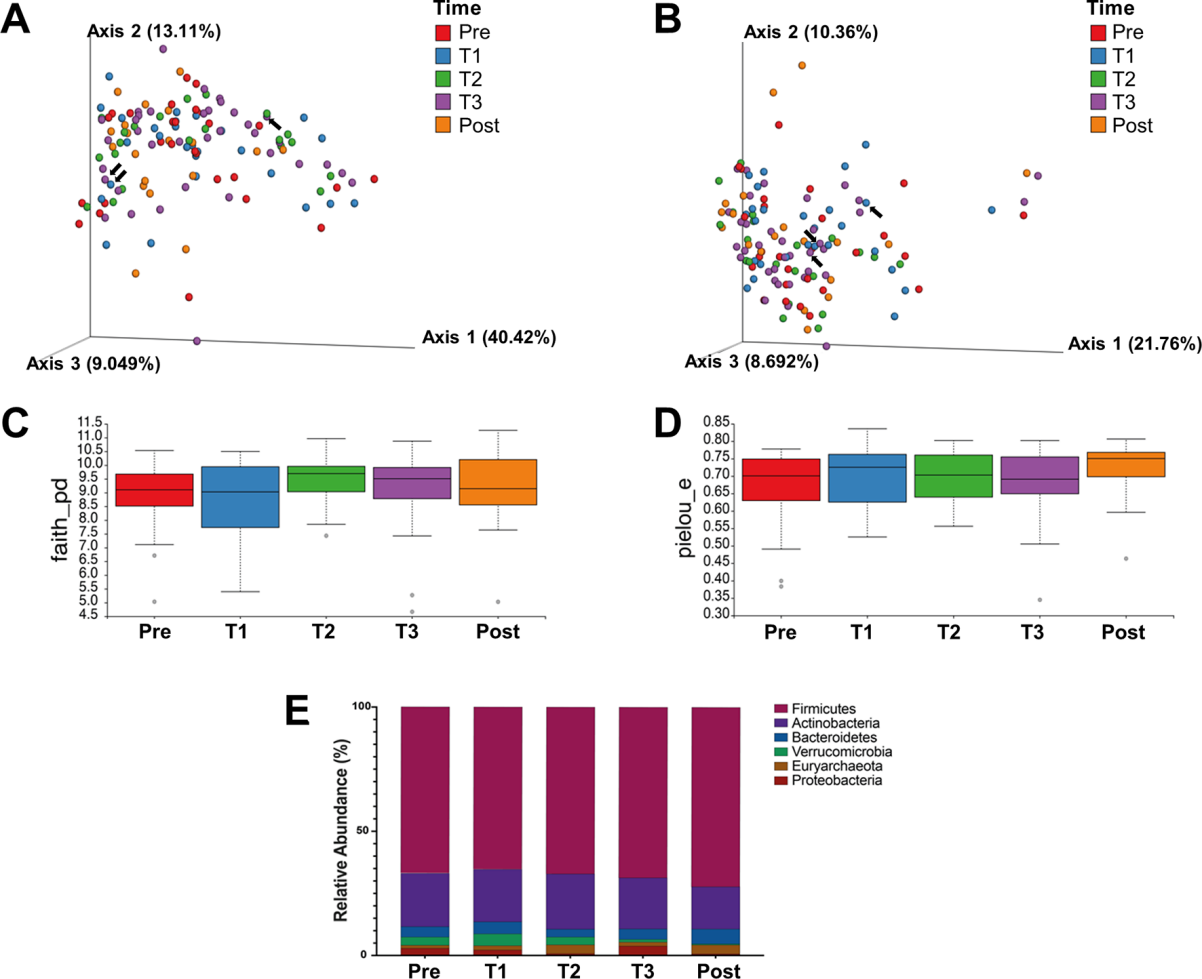
Microbial diversity parameters of patients with IBD do not change throughout pregnancy. Faecal samples from patients with IBD were collected at five time points: prepregnancy, first, second, third trimester and postpartum (27, 27, 21, 36 and 19 samples, respectively). (A and B) β-diversity using principal coordinate analysis of unweighted (A) and weighted (B) UniFrac distances. The black arrows point to samples of patients who used antibiotics. (C and D) α-Diversity using Faith’s phylogenetic diversity (C) and Pielou’s evenness plot (D) measurements. (E) Taxonomy plot at the phylum level.

We then analysed the microbiota of all patients with IBD throughout pregnancy. Beta diversity (between sample) analysis did not reveal any significant differences relating to the different time points or use of antibiotics (figure 3A, B). The richness (figure 3C) and evenness (figure 3D) as measured by Faith’s PD and Pielou respectively also did not differ significantly over time. When looking at the relative abundance (figure 3E), Firmicutes tended to increase as pregnancy progressed and Actinobacteria and Verrucomicrobia decreased, although not significantly.

**Figure 4:**
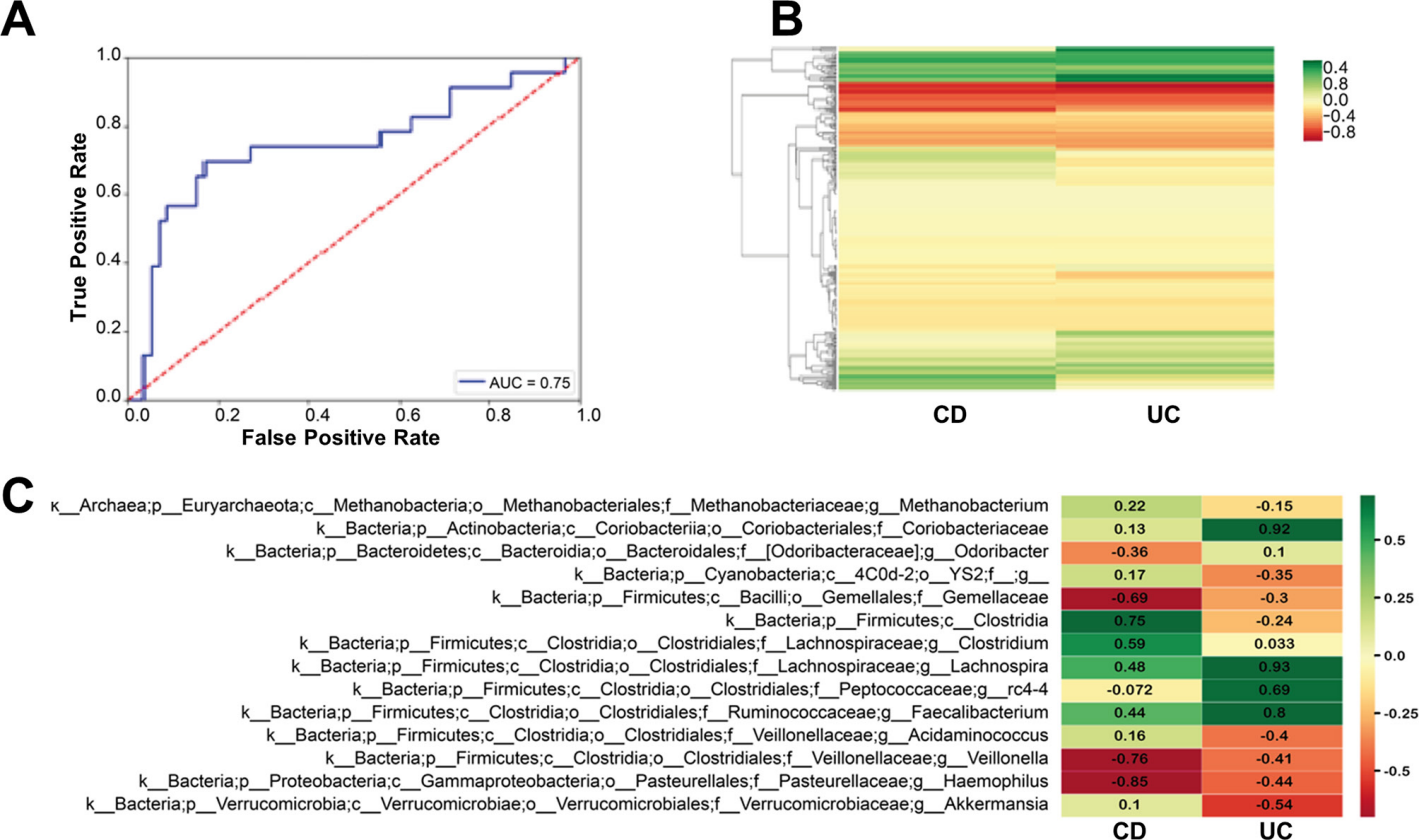
Overall microbiome composition of patients with CD differs from patients with UC. (A) ROC curve of classification of UC (n=41) versus CD (n=89). (B) Spectral clustering of bacteria based on the bacterial differences between the UC and CD. (C) Bacteria with differences between UC and CD with significance with p<0.05. The marked values are the average (^10^log) value for each bacterium in each group. CD, Crohn’s disease; ROC, receiver operating characteristic.

Figure 4 summarises the differences between women with CD and women with UC. When classifying patients with CD and UC, the area under curve was 0.75 for the test results, showing that the microbiome reflects disease type (figure 4A). Spectral clustering of the bacteria demonstrated differences in bacterial communities between patients with CD and UC (figure 4B) and a more specific analysis showed significant differences in several bacteria that behaved differently between the two diseases (figure 4C). For example, *Methanobacterium*, *Acidaminococcus*and *Akkermansia*, an unclassified member of the YS2 order and an unclassified member of the Clostridia class, were all positively correlated with the CD microbiome and negatively correlated with the UC microbiome. *Odoribacter* and an unclassified member the family Peptococcaceae showed the opposite behaviour (figure 4C).

**Figure 5:**
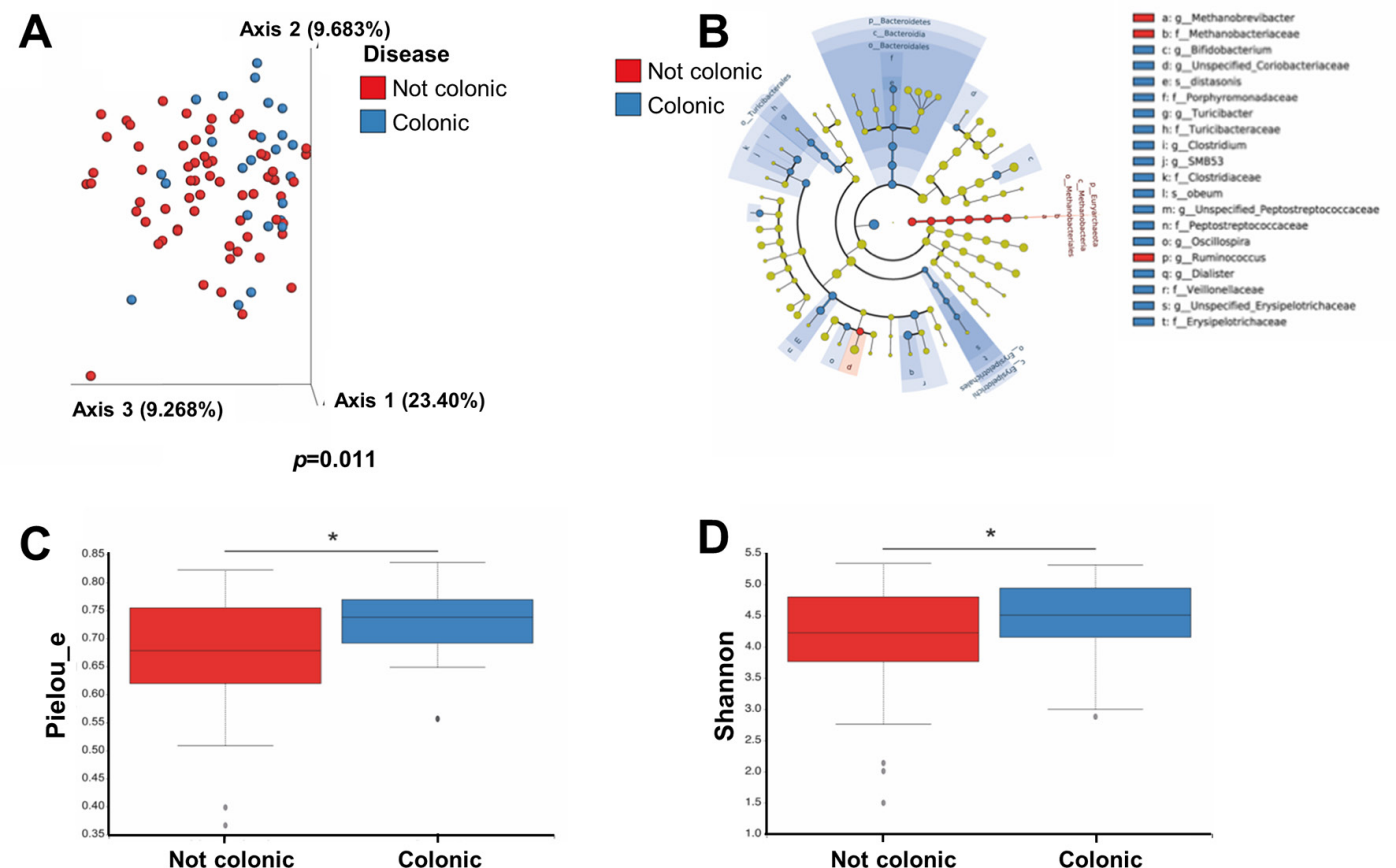
The microbiome of patients with CD suffering from colon disease is different than that of patients with non-colonic disease. Patients with CD were divided according to disease location: colonic (n=24) and not colonic (n=65). (A) β-diversity using principal coordinate analysis of unweighted UniFrac distances (p=0.011) (B) Significantly abundant taxa in each of the groups by LEfSe analysis. (C and D) α-diversity using (C) Pielou’s evenness plot (p=0.02) and (D) Shannon’s diversity index (p=0.043). LEfSe, linear discriminant analysis effect size.

Having established that UC and CD patients’ microbial signatures are distinctive, we next asked whether disease location in patients with CD would also affect microbial composition. Unweighted UniFrac demonstrated significant differences in faecal microbiome between patients suffering from colonic versus non-colonic disease (p=0.011) (figure 5A). In addition, we found significant feature differences. In patients with exclusive colonic disease, features such as *Bifidobacterium*, *Turicibacter*, *Clostridium*, *Oscillospira* and *Diallster* were highly abundant. In the patients with ileal or ileocolonic disease, the features *Methanobrevibacter* and *Ruminococcus* were more abundant (figure 5B). Alpha diversity was higher in samples from patients with colonic disease by using Pielou’s Evenness plot (p=0.02) and Shannon’s diversity index (0.043) (figure 5C, D). Colonic disease in patients with CD is not similar to UC disease: significant differences were seen when comparing colonic and non-colonic CD patient samples to UC samples (unweighted UniFrac beta diversity differences, p*=*0.035 and p=0.028, respectively, data not shown).

**Figure 6:**
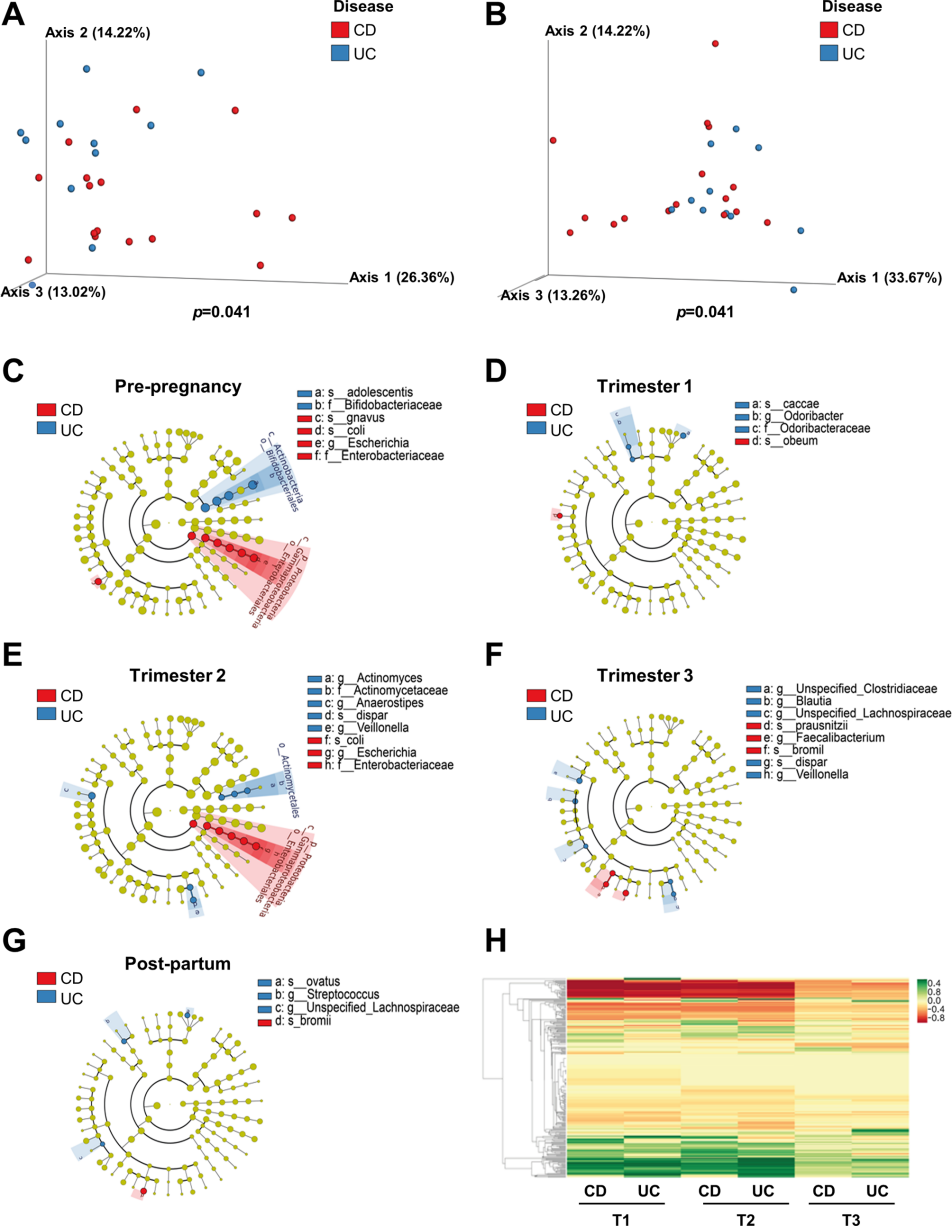
The microbiomes of patients with CD and UC are dominated by different species at each time point. (A and B) β-diversity using principal coordinate analysis of unweighted (A) and weighted (B) UniFrac distances (p=0.041) of prepregnancy samples (CD n=16, UC n=11). (C–G) Cladogram of significant differentially abundant microbial taxa obtained using LEfSe of prepregnancy (C), first (CD n=19, UC n=8) (D), second (CD n=16, UC n=5) (E), third (CD n=25, UC n=11) (F) trimester and postpartum (CD n=13, UC n=6) (G) gut microbiomes. (H) Spectral clustering of bacteria based on the difference between the UC and CD and over all trimesters. CD, Crohn’s disease; LEfSe, linear discriminant analysis effect size.

When comparing the differences between patients with UC and CD at a given time point, significant differences in beta diversity were observed in the prepregnancy samples (p=0.041) in unweighted and weighted UniFrac (figure 6A, B), but not at T1, T2 or T3 or postpregnancy (data not shown). LEfSe analysis, which is based on LDA scores highlighted the significant features at each time point (figure 6C–G). Before initiation of pregnancy, women with UC had higher abundance of *Bifidobacterium adolescentis* compared with women with CD, who had higher abundances of *Ruminococcus gnavus* and *Escherichia coli* (figure 6C). In early pregnancy, women with UC had an over-representation of *Bacteroides caccae* and the genus *Odoribacter*, whereas women with CD had increased levels of *Blautia obeum* (figure 6D). The higher levels of *E. coli* seen in prepregnant women with CD appeared again in T2. Women with UC had higher levels of the genera *Actinomyces*, *Anaerostipes* and *Veillonella* (figure 6E). *Veillonella* remained significantly higher in these women in T3 as well, and this was accompanied with higher abundance *Blautia* and unclassified members of the Clostridiaceae and Lachnospiraceae compared with the CD microbiome in T3, which had higher levels of *F. prausnitzii* and *Ruminococcus bromii* (figure 6F). *R. bromii* remained over-represented postpregnancy too (in CD patients), as opposed to *Bacteroides ovatus*, Streptococcus and an unclassified member of Lachnospiraceae (higher in T3 as well) that increased postpartum in women with UC (figure 6G). A centroid-based clustergram (figure 6H) allows for visualisation of the differences between the two IBD states and of those observed at the different time points related to pregnancy.

### The effect of inflammation and medication on microbial signatures during pregnancy in IBD

**Figure 7:**
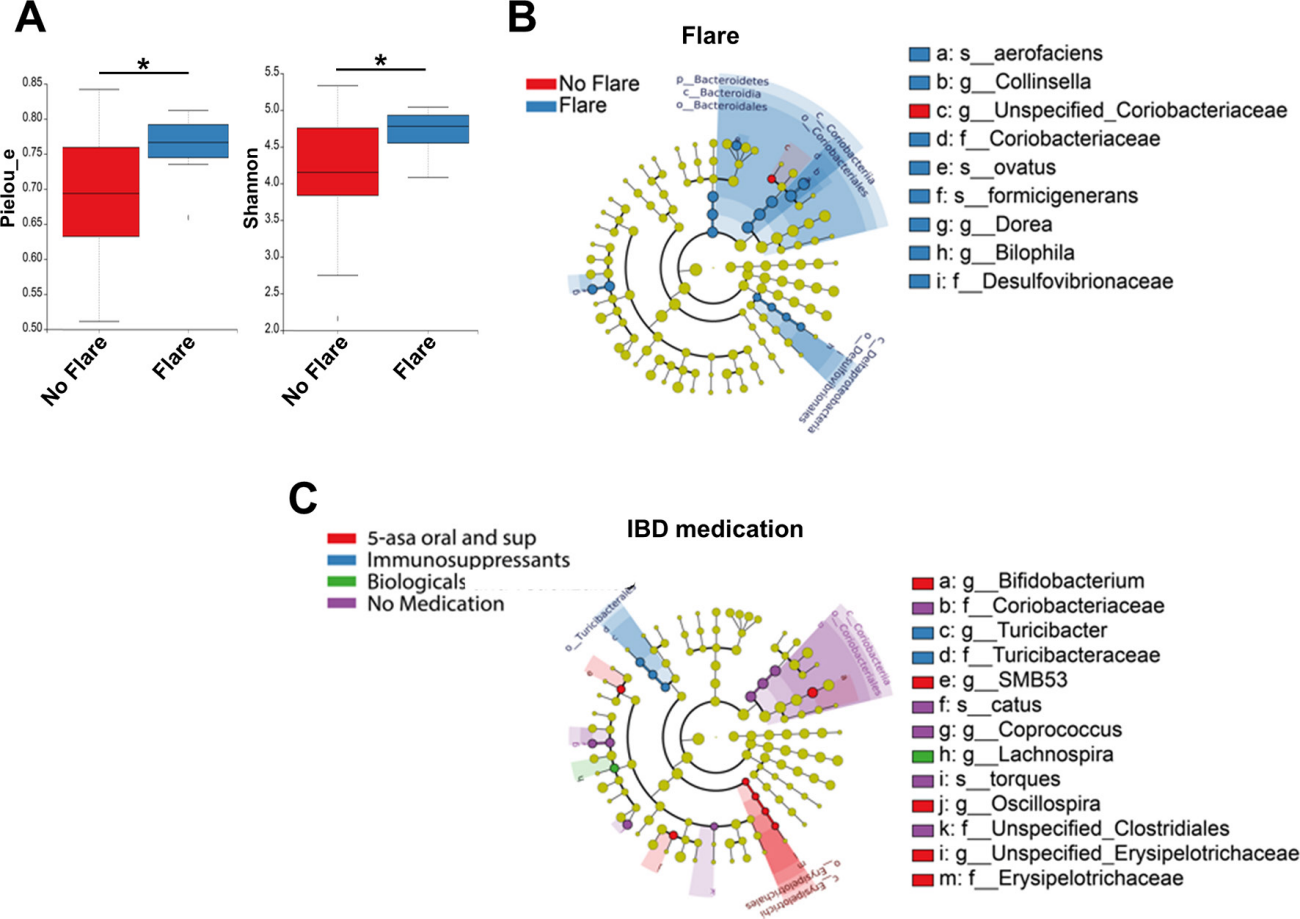
Microbiota of patients with CD differs when suffering from a flare. Patients were divided according to those who suffered a flare (eight samples) during gestation compared with those with stable disease (51 samples) and by IBD medication: 5-ASA oral and sup, immunosuppressants, biologicals and no medication (3, 14, 12 and 23 samples, respectively). (A) α-Diversity using Pielou’s evenness plot (p=0.025) and Shannon’s diversity index measurements (p=0.03) comparing flare and no flare samples. (B and C) Cladogram of significantly differentially abundant microbial taxa obtained using LEfSe divided by flare occurrence (B) or IBD medication (C). CD, Crohn’s disease.

Differences in the microbiome of patients that experienced a flare during pregnancy compared with those who did not were also analysed for patients with CD and UC separately. Pregnant women with CD had significantly higher bacterial evenness (p=0.025) and richness (p=0.03, figure 7A) when experiencing a flare. LEfSe analysis demonstrated several features that were over-represented in women with a flare. These included *Collinsella aerofaciens*, *Bacteroides ovatus*, *Dorea formicigenerans*, *Bilophila* and the phylum Bacteroidetes in general (figure 7B). For patients with UC, no significant differences were found in richness and evenness measurements (online figure S4A, B), although LEfSe analysis indicated that women suffering from a flare had higher relative abundance of *Odoribacter*, *Bilophila* and *Parabacteroides distasonis*, whereas the microbiome of women who did not suffer from a flare was enriched with *Coprococcus*, *Lachnospira* and *F. prausnitzii*.

10.1136/gutjnl-2019-318263.supp5 Supplementary data

Next, we analysed the effect different medications had on the microbiome. LEfSe analysis revealed multiple features that were different between the different treatments, which are summarised in figure 7C. Interestingly, *Coprococcus catus* and *Ruminococcus torques* were over-represented in women with CD who did not receive any medication. No significant differences were observed for different treatments in patients with UC. We also examined the effect of BMI and did not observe any influence on β-diversity or α-diversity. However, we did find two taxa that differed (*Dorea formicigenerans* was more overrepresented in BMI <25 and *R. bromii* was overrepresented in BMI >25, data not shown).

### Parity influences the microbiome in pregnant patients with IBD

**Figure 8:**
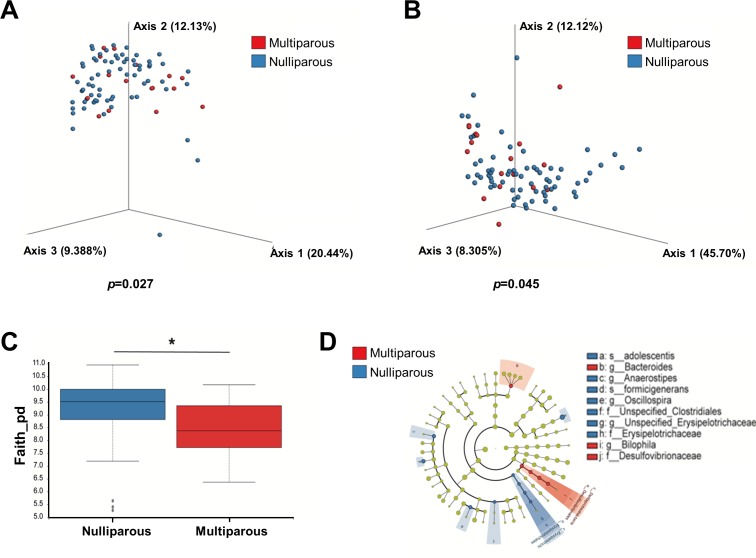
The microbiome of multiparous pregnancies is different from nulliparous pregnancies. Faecal samples collected during gestation were divided according to patients’ previous pregnancies into nulliparous (n=67) versus multiparous (n=17). (A and B) β-diversity using principal coordinate analysis of unweighted (A) and weighted (B) UniFrac distances (p=0.027 and p=0.045, respectively). (C) α-Diversity using Faith’s phylogenetic diversity (p=0.017). (D) Differently abundant taxa in each of the groups by LEfSe analysis.

The microbiome of multiparous women with IBD compared with nulliparous women exhibited significant differences in beta diversity in unweighted and weighted UniFrac (p=0.027 and p=0.045, respectively) (figure 8A, B). In addition, nulliparous patients had higher bacterial richness (figure 8C). Several features were highly abundant in nulliparous samples as found by LEfSe analysis such as *Anaerostipes* and *Oscillospira*. However, *Bacteroides* and *Bilophila* were more abundant in multiparous patients (figure 8D). No differences were seen on cytokine patterns between IBD women who had or who had not born children before (not shown).

### The microbiome from pregnant patients with IBD is less rich and more similar than microbiome of pregnant healthy controls

**Figure 9:**
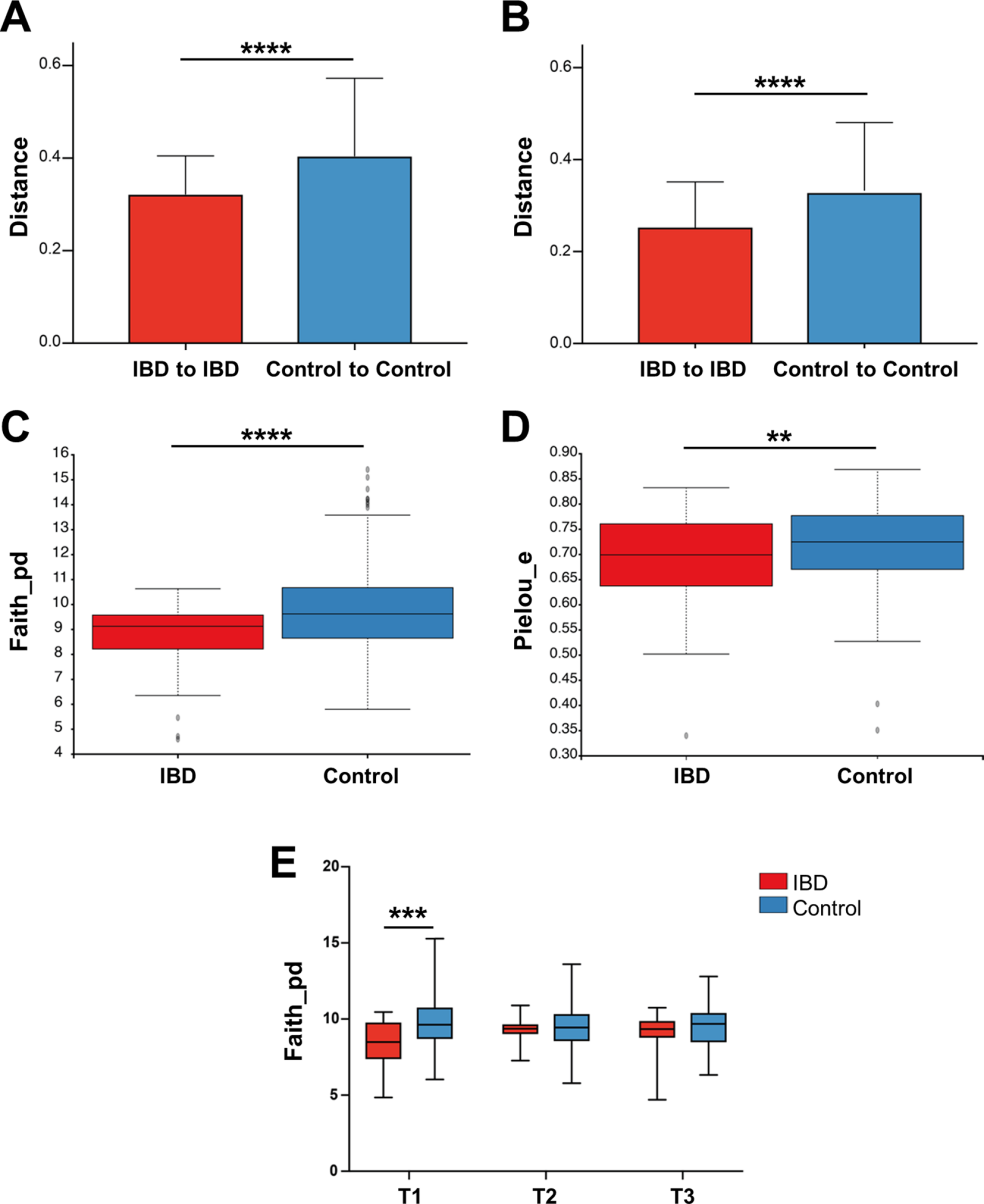
Patients with IBD have more uniform microbiomes than healthy controls. A comparison of the gut microbiomes of IBD (130 samples) and control (236 samples) pregnant women. (A and B) Beta-diversity of unweighted (A) and weighted (B) (p=0.001) UniFrac distances. (C and D) α-Diversity using (C) Faith’s phylogenetic diversity (p<0.0001), and (D) Pielou’s evenness plot (p=0.008) measurements. (E) Faith’s phylogenetic diversity comparing IBD and control samples by pregnancy trimesters (p=0.0004).

Since the two cohorts originated from different countries and were extracted using different kits, we only compared diversity indices as we have shown previously that this to be a valid strategy.^19^ The microbiota of patients with IBD was more similar to one another (beta diversity) both by unweighted (figure 9A; p=0.001) and weighted UniFrac (figure 9B; p=0.001) than that of healthy controls. Patients with IBD also had lower bacterial richness as measured by Faith’s PD (figure 9C; p<0.001) and evenness as measured by Pielou’s evenness (figure 9D; p=0.008). We also compared the alpha diversity differences per trimester. Patients with IBD showed a significantly reduced bacterial richness in T1 compared with the controls (figure 9E; p=0.001), which may even be underestimated, as patients with IBD were also more often nulliparous and nulliparity was associated with higher alpha diversity in these patients. This effect disappeared later in pregnancy. Exclusion of comorbidities did not alter these results.

### Cytokine microbial network and dynamic analysis

**Figure 10:**
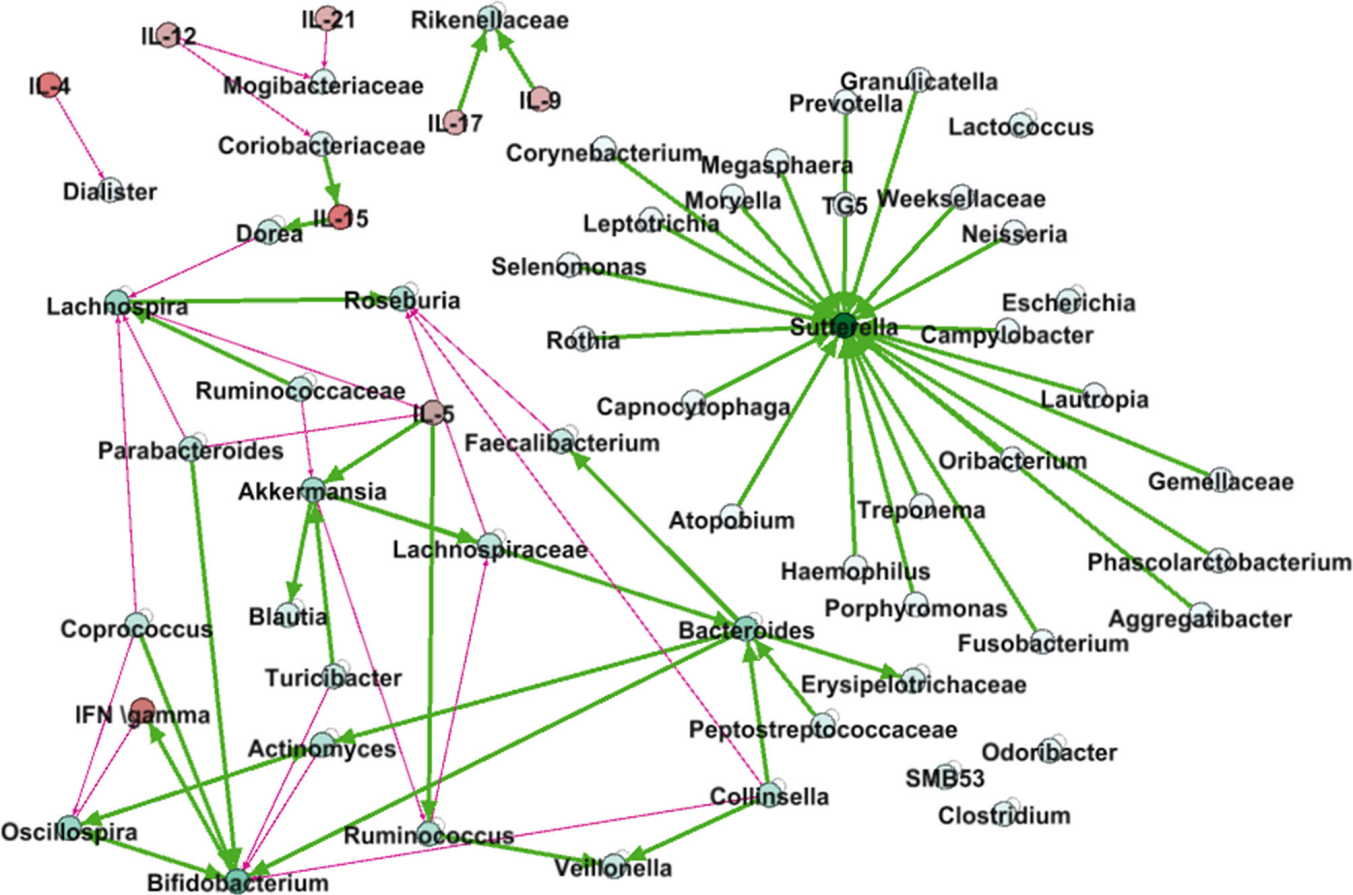
Correlation between current value of the source node and the change in the value of a target node. Thick green arrowsrepresent positive correlations, while thin red arrowsrepresent negative correlations. A thin grey round arrow represents a correlation between the current value of a feature and future change. Cytokines are marked in red nodes.

As summarised in figure 10, *Sutterella* was the hub of a bacterial network. This means that the abundance of multiple features was positively correlated to an increase in the abundance of *Sutterella* at a later timepoint. We also observed negative correlations between bacteria, for example, the abundance of *Faecalibacterium* was correlated to a decrease in abundance of *Roseburia* at a second timepoint. When modelling the interactions between levels of cytokines and bacterial abundance, we again observed positive and negative correlations. For example, the levels of IL-9 and IL-17 were both correlated with a decrease in the abundance of an unclassified genus of the Rikenellaceae, whereas IL-5 was positively correlated with an increase in the abundance of *Akkermansia* and *Ruminococcus*. Overall, these analyses provide a wealth of data detailing the correlations between microbiome, disease type, cytokine profiles and pregnancy.

## Discussion

Normal pregnancy is associated with hormonal, microbial and immunological changes, which prepare the maternal body for successful childbirth. Interestingly, some autoimmune diseases, including rheumatoid arthritis and multiple sclerosis, show improvement during pregnancy, while risk of flares increases postpartum.^14–16^ This suggests that pregnancy-induced physiological changes affect immune processes at peripheral sites, and it has been suggested that increased levels of Tregs and a shift towards Th2 cytokine patterns contribute to amelioration of Th1-driven diseases^41 42^ and conversely also play a role in exacerbation of Th2-driven inflammatory diseases such as systemic lupus erythematosus during pregnancy.^43 44^ CD is generally thought to constitute a Th1/Th17 disease, while UC is more Th2/Th17 driven, which may explain differences in disease patterns during pregnancy observed between these types of IBD.^10^ However, while surprisingly little consensus has been reached for other cytokines, one of the most consistent findings in healthy women are rising peripheral blood levels of IL-6 (generally considered a Th1 cytokine) from early to late pregnancy.^18 45–47^ Our own results confirm that changes in cytokine patterns from T1 to T3 are present during healthy pregnancy. However, it was not possible to ascribe our observed changes during pregnancy to either Th2 cytokines (eg, IL-4, IL-5 and IL-13) or cytokines commonly associated with Th1 responses (IFNγ and TNFα). While we observed increased levels of IL-6 and reduced expression of TNFα during late gestation in healthy females, both of these have been ascribed to both Th1 and Th2 cells as well as a range of other cell types.^48^ Thus, based on our data, it is unlikely that an outspoken Th2 shift during pregnancy ameliorates (auto-)immune diseases. However, in the current study, we do show that several proinflammatory cytokines (IL-6, IL-8, IL-12, IL-17 and TNFα), known to play a role in IBD pathophysiology,^49 50^ decrease significantly on conception, suggesting that pregnancy reduces immunological parameters of inflammation in patients with IBD. During pregnancy itself, serum cytokine levels in patients with IBD subsequently remained relatively stable, with reduced levels of IL-5 and IL-10 levels and increased IL-8 and IFNγ levels compared with control, throughout the three trimesters. Overall, it seems that the immunological state of patients with IBD improves on pregnancy.

Differences between CD and UC microbiomes have been reported previously,^51^ and accordingly in this study, we could differentiate between CD and UC solely based on the microbiomes with an area under the curve (AUC) of 0.75. Here, we studied the effect of pregnancy on the UC and CD microbiomes as we know that pregnancy influences the microbiota. The microbiomes of patients with CD and UC remained different from each other throughout pregnancy. However, while before pregnancy, beta diversity differed between patients with UC and CD, the onset of pregnancy caused a shift in beta diversity, which caused the microbiomes to behave similarly diversity wise. In general, it seems that as pregnancy progressed changes in bacterial features became more subtle (figure 6H), suggesting a dampening effect of pregnancy on microbial differences. Indeed very few patients experienced relapse of disease during pregnancy. Those patients with CD experiencing a flare had a significantly less diverse and evenly distributed microbiome than patients with CD who did not experience a flare. At the genus level, the only genus increased in both patients with CD and UC who suffered from a flare at any point during pregnancy was *Bilophila*, which has been shown to increase under inflammatory and pathological conditions such as inflammatory disorders and appendicitis^52^ and has been suggested to be involved in the initiation of IBD.^53^ However, the butyrate producing *F. prausnitzii* was over-represented in patients with UC with no relapse. *F. prausnitzii* is considered to have anti-inflammatory properties which may help dampen the flaring process and has even been considered to have a clinical potential in IBD.^54^

Two of the main microbiome characteristics observed in both disease and pregnancy are lower alpha diversity and greater beta diversity.^19 55^ The comparison of IBD with healthy microbiomes revealed that the IBD microbiomes were less diverse and even than the healthy controls. This trend of lower diversity in patients with IBD has been previously reported^51 56^ and was expected. To our surprise, we observed that the IBD microbiomes were more similar to one another (lower beta diversity), suggesting that the same species are disappearing during disease from the majority of patients. We could not identify which bacteria differed between the two cohorts as the two cohorts are from different countries and the DNA was isolated using different protocols, and we have previously shown that samples extracted via different methods still show the same diversity patterns but might change at feature levels.^19^ Nevertheless, we have previously demonstrated that during pregnancy in healthy females microbial diversity decreases.^19^ The fact that alpha diversity differed between patients with IBD and controls during early pregnancy but decreased at later gestational times indicates that pregnancy in IBD is not followed by an additional loss of diversity on top of the already altered microbial composition in these patients.

In order to produce a dynamic model, we tested the correlation between changes in cytokine or bacteria log levels between two time points and the value of all cytokines and log bacteria expression in the initial time. While these correlations are not a clear evidence of causality since common cause effects can ruin causality, they provide a first order dynamic model. Of great interest was *Sutterella*, which increased in correlation to several other bacteria. *Sutterella* has previously been shown to be increased in patients with IBD compared with healthy controls.^57^ The negative correlation between *Oscillospira* and IFNγ has been reported previously in mice.^58^ Oscillospira is a known butyrate producer and has been shown to be decreased in inflammatory states.^59^ The interaction between *Oscillospira* and *Actinomyces* is also worth mentioning as the first was described to be decreased in IBD,^57^ whereas the latter has been shown to increase in patients with IBD.^60^ The association between the abundances of *Faecalibacterium* and *Roseburia* and IBD was also described previously,^61^ but our model demonstrates that the abundance of *Faecalibacterium* is associated with the decrease in abundance of *Roseburia*.

Thus, in toto, these data suggest that immunological parameters improve in patients with IBD on pregnancy, while microbial diversity normalises to that seen in healthy pregnancy.
