# Supplementary material for: Modulation of cytokine patterns and microbiome during pregnancy in IBD
Source: Gut. 2019 Jun 5;69(3):473–86. doi: 10.1136/gutjnl-2019-318263 (PMC7034354; doi:10.1136/gutjnl-2019-318263)
Supplement: Supplementary data [file gutjnl-2019-318263supp001.pdf]

Table S1 Patient characteristics (fecal samples)

		Pre-pregnancy	T1	T2	T3	Post-partum
Mean age at diagnosis (y, SD)		22.6 (3.7)				
weeks pregnant at collection (SD)			10.3 (3)	19.7 (3)	31.9 (3.3)	
IBD diagnosis (%)	CD<br>UC	15 (32.6)<br>31 (67.4)				
Disease location CD (Montreal) (%)	L1 Ileal<br>L2 Colonic<br>L3 Ileocolonic	5 (16.1)<br>8 (25.8)<br>18 (58.1)				
Disease behavior CD (Montreal) (%)	B1 non structuring non penetrating<br>B2 Stricturing<br>B3 Penetrating<br>B2+B3 Stricturing and penetrating	23 (74.2)<br>2 (6.5)<br>4 (12.9)<br>2 (6.5)				
Disease extent UC/IBD-U (Montreal) (%)	E1 Proctitis<br>E2 Left-sided colitis<br>E3 Pancolitis	2 (13.3)<br>7 (46.7)<br>6 (40)				
Median HBI for CD (IQR)			1 (0-2.5)	0.5 (0-2)	0 (0-2.5)	
Median SCCAI for UC/IBD-U (IQR)			1 (04)	1 (0.5-4.5)	1 (0.5-1.5)	
Median disease duration (y, IQR)		5 (2-9)				
IBD maintenance medication (monotherapy)	None<br>5-ASA<br>Steroids<br>Thiopurine<br>Ani-TNF/anti-integrin	9 (109.6)<br>7 (15.2)<br>1 (2.2)<br>8 (17.4)<br>13 (28.3)	11 (23.9)<br>4 (8.7)<br>1 (2.2)<br>7 (15.2)<br>12 (26.1)	11 (23.9)<br>4 (8.7)<br>0<br>7 (15.2)<br>12 (26.1)	20 (43.5)<br>4 (8.7)<br>1 (2.2)<br>9 (19.6)<br>4 (8.7)	10 (21.7)<br>6 (13)<br>0<br>7 (15.2)<br>12 (26.1)
IBD maintenance medication (combination)	Anti-TNF + thiopurine<br>Anti-TNF + thopurine + 5-ASA<br>Anti-TNF + steroids<br>Anti-TNF + steroids + 5-ASA<br>Thiopurine + 5-ASA<br>5-ASA + steroids	3 (6.5)<br>0<br>1 (2.2)<br>2 (4.3)<br>2 (4.3)<br>0	2 (4.3)<br>2 (4.3)<br>2 (4.3)<br>0<br>3 (6.5)<br>2 (4.3)	2 (4.3)<br>2 (4.3)<br>2 (4.3)<br>0<br>3 (6.5)<br>2 (4.3)	0<br>1 (2.2)<br>1 (2.2)<br>0<br>3 (6.5)<br>2 (4.3)	2 (4.3)<br>2 (4.3)<br>2 (4.3)<br>0<br>3 (6.5)<br>2 (4.3)
IBD surgery (%)	Bowel resection<br>Perianal surgery<br>Pouch	6 (13)<br>2 (4.3)<br>1. (2.2)				

	Appendectomy	1 (2.2)				
Flare (%)		3 (6.5)	5 (10.9)	6 (13)	3 (6.5)	3 (6.5)

CD: Crohn's disease; UC: ulcerative colitis; CD: standard deviation; IQR: interquartile range; y: years; IBD: inflammatory bowel disease; HBI: Harvey Bradshaw index; SCCAI: simple clinical colitis activity index; TNF: tumor necrosis factor; ASA: aminosalicylic acid;

Table S2 Patient characteristics (serum samples)

		Pre-pregnancy	T1	T2	T3	Post-partum
Mean age at diagnosis (y, SD)		22.8 (4.9)				
weeks pregnant at collection (SD)			8.4 (2.1)	21.1 (2.6)	32 (3)	
IBD diagnosis (%)	CD<br>UC	8 (24.2)<br>25 (75.8)				
Disease location CD (Montreal) (%)	L1 Ileal<br>L2 Colonic<br>L3 Ileocolonic	2 (8)<br>11 (44)<br>12 (48)				
Disease behavior CD (Montreal) (%)	B1 non structuring non penetrating<br>B2 Stricturing<br>B3 Penetrating<br>B2+B3 Stricturing and penetrating	16 (64)<br>2 (8)<br>3 (12)<br>4 (16)				
Disease extent UC/IBD-U (Montreal) (%)	E1 Proctitis<br>E2 Left-sided colitis<br>E3 Pancolitis	1 (12.5)<br>3 (37.5)<br>4 (50)				
Median HBI for CD (IQR)			3 (1-5.5)	1 (0-3.5)	1 (0-2.5)	
Median SCCAI for UC/IBD-U (IQR)			4 (0.505.5)	4 (0-5)	1 (1-2)	
Median disease duration (y, IQR)		6.6 (4.8)				
IBD maintenance medication (monotherapy)	None<br>5-ASA<br>Thiopurine<br>Ani-TNF/anti-integrin	3 (9.1)<br>7 (21.1)<br>3 (9.1)<br>15 (45.5)	6 (18.2)<br>5 (15.2)<br>2 (6.2)<br>15 (45.5)	6 (18.2)<br>5 (15.2)<br>2 (6.1)<br>16 (48.5)	18 (54.5)<br>5 (15.2)<br>3 (9.1)<br>3 (9.1)	6 (18.2)<br>5 (15.2)<br>2 (6.1)<br>16 (48.5)
IBD maintenance medication (combination)	Anti-TNF + thiopurine<br>Anti-TNF + thopurine + 5-ASA<br>Anti-TNF + steroids<br>Anti-TNF + steroids + 5-ASA<br>Thiopurine + 5-ASA<br>5-ASA + steroids	1 (3)<br>2 (6.1)<br>1 (3)<br>1 (3)<br>0<br>0	1 (3)<br>1 (3)<br>1 (3)<br>0<br>1 (3)<br>1 (3)	1 (3)<br>0<br>1(3)<br>0<br>1 (3)<br>1 (3)	0<br>1 (3)<br>1 (3)<br>0<br>1 (3)<br>1 (3)	2 (3)<br>0<br>1 (3)<br>0<br>1 (3)<br>1 (3)
IBD surgery (%)	Bowel resection<br>Perianal surgery<br>Pouch<br>Appendectomy	5 (15.2)<br>4 (12.1)<br>1 (3)				

Flare (%)		4 (12.1)	6 (18.2)	6 (15.2)	3 (9.1)	2 (6.1)

CD: Crohn's disease; UC: ulcerative colitis; CD: standard deviation; IQR: interquartile range; y: years; IBD: inflammatory bowel disease; HBI: Harvey Bradshaw index; SCCAI: simple clinical colitis activity index; TNF: tumor necrosis factor; ASA: aminosalicylic acid;
