# Supplementary figures and images for: Modulation of cytokine patterns and microbiome during pregnancy in IBD
Source: Gut. 2019 Jun 5;69(3):473–86. doi: 10.1136/gutjnl-2019-318263 (PMC7034354; doi:10.1136/gutjnl-2019-318263)

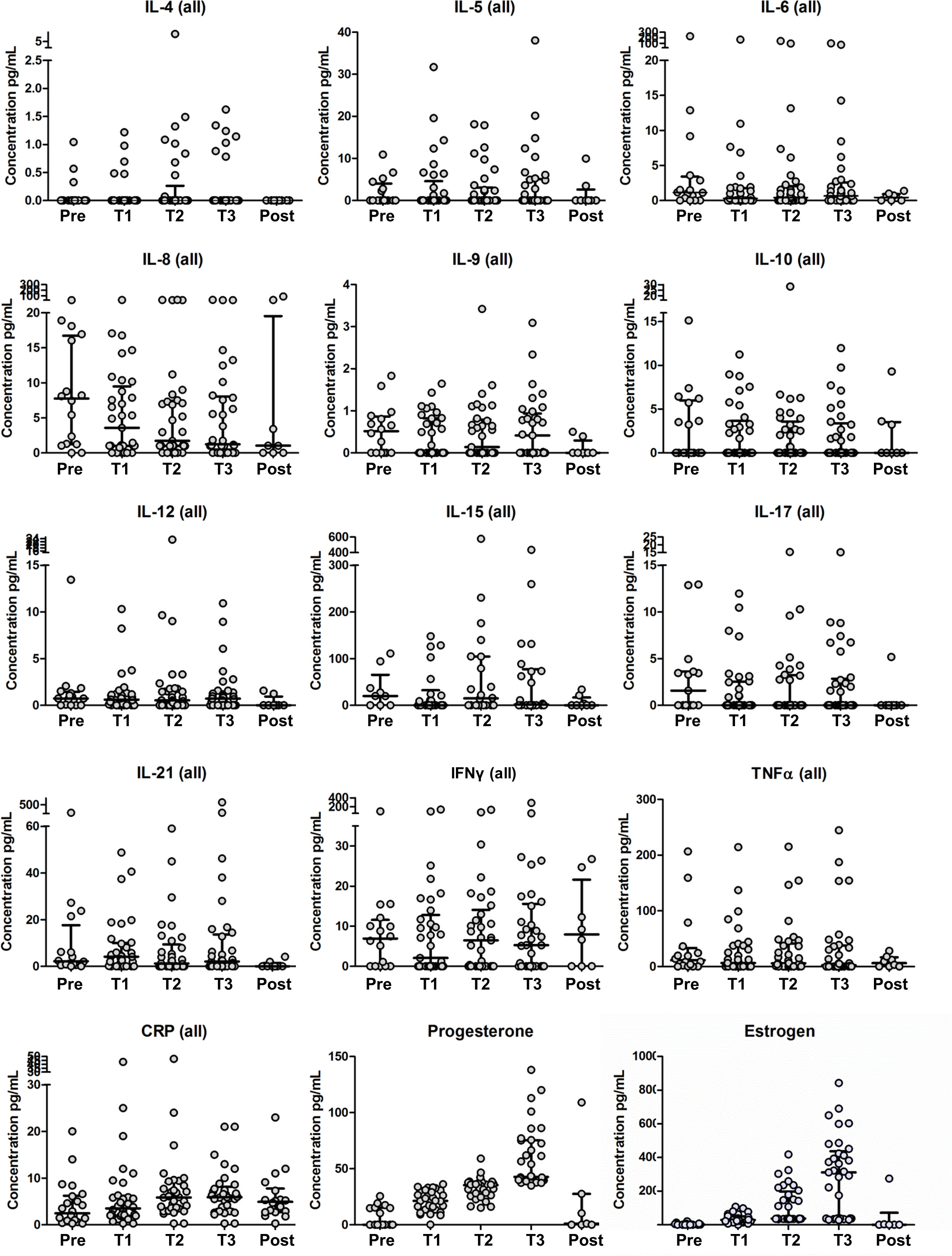

Supplement: Supplementary data [file gutjnl-2019-318263supp002.jpg]

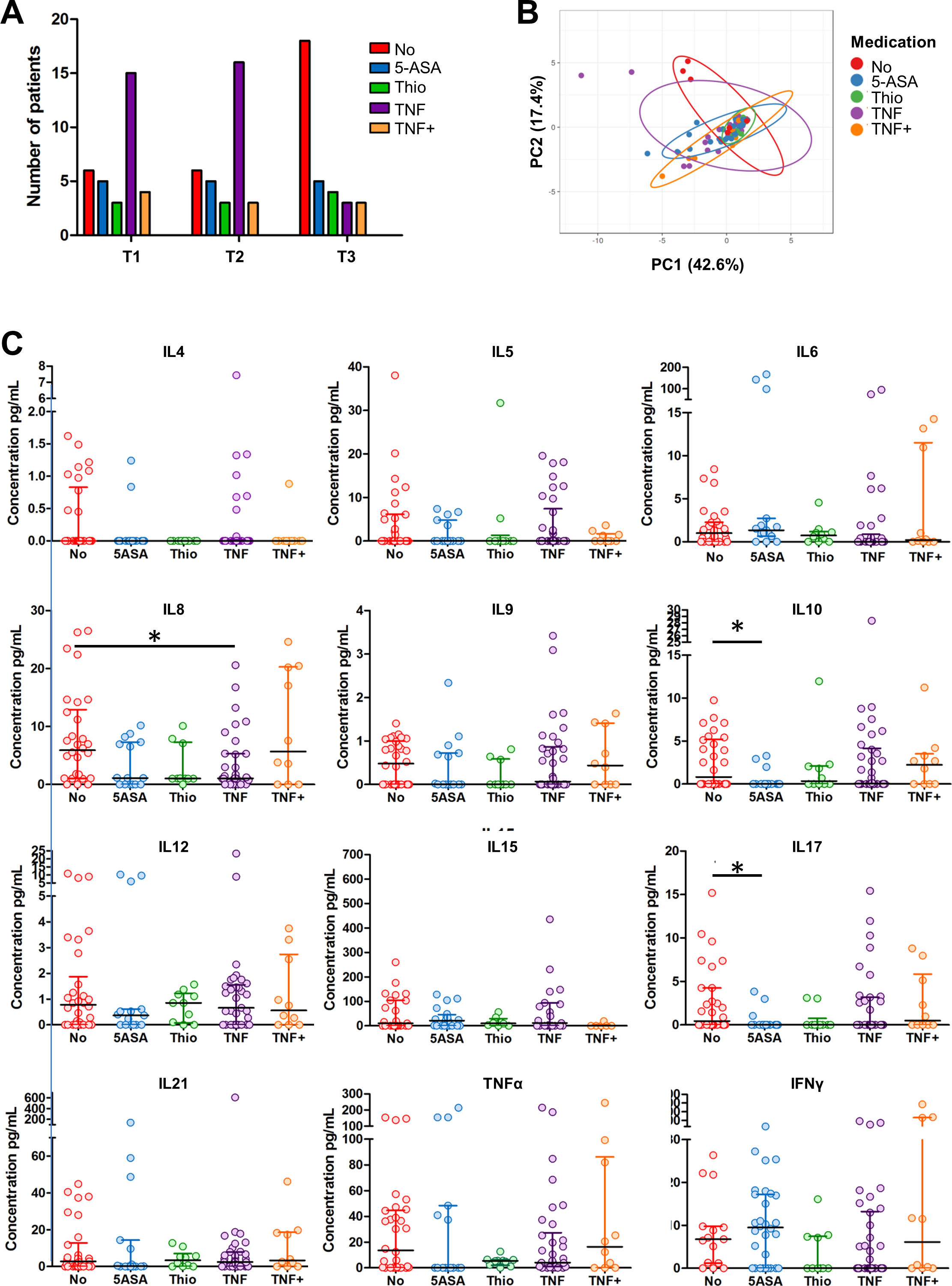

Supplement: Supplementary data [file gutjnl-2019-318263supp003.jpg]

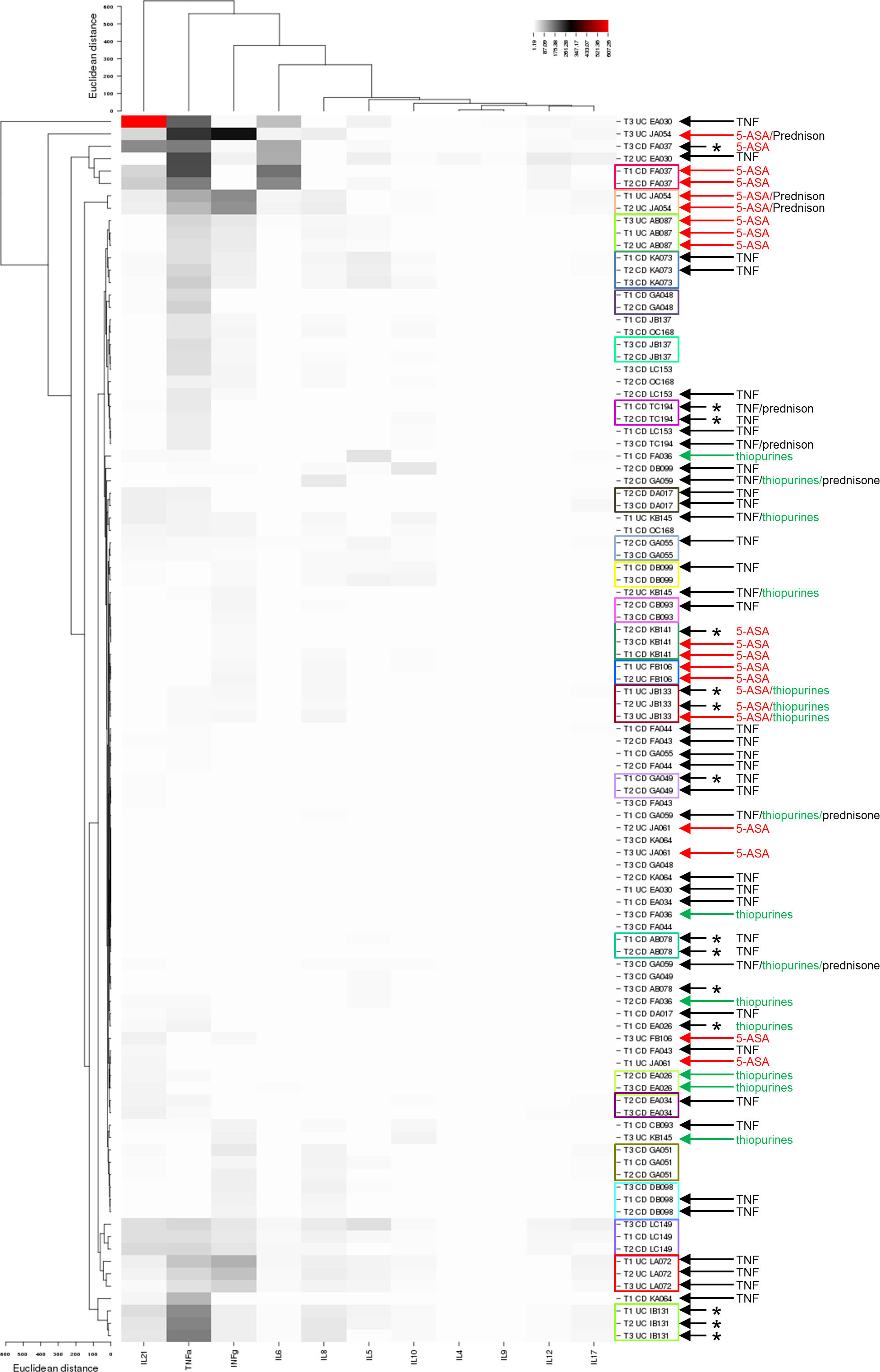

Supplement: Supplementary data [file gutjnl-2019-318263supp004.jpg]

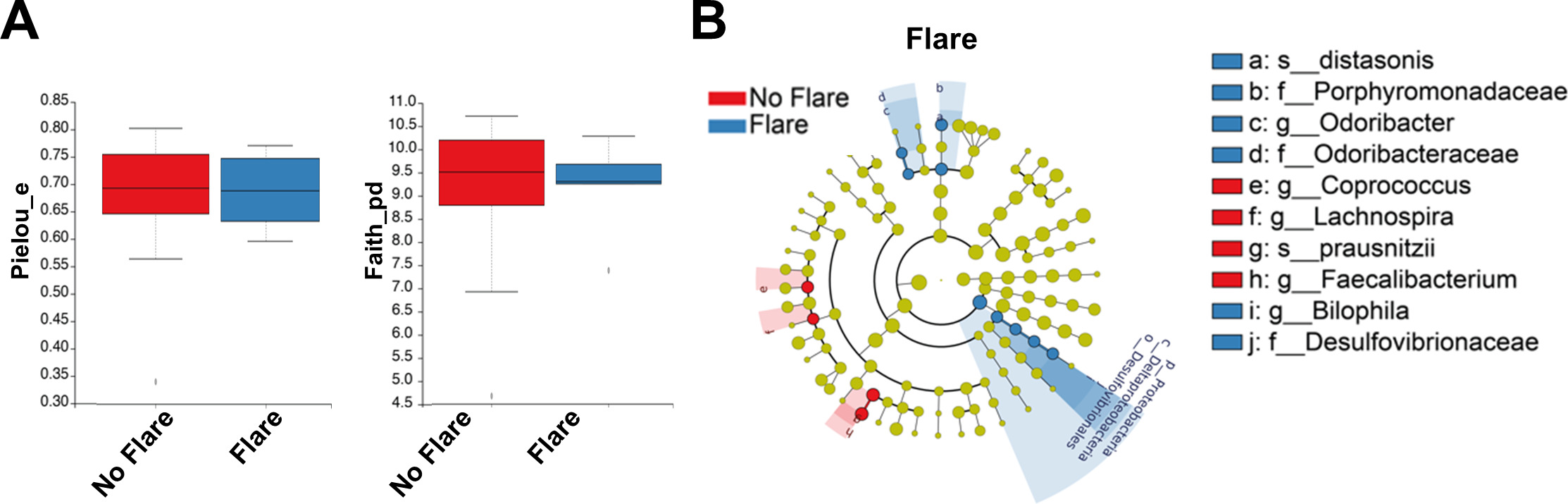

Supplement: Supplementary data [file gutjnl-2019-318263supp005.jpg]
